# Croatian Action on Salt and Health (CRASH): On the Road to Success—Less Salt, More Health

**DOI:** 10.3390/nu16101518

**Published:** 2024-05-17

**Authors:** Bojan Jelaković, Mihaela Marinović Glavić, Marija Batinić Sermek, Lovorka Bilajac, Marija Bubaš, Vlatka Buzjak Služek, Krunoslav Capak, Ines Drenjančević, Andrea Gross Bošković, Ana Jelaković, Tomislav Jukić, Sanja Kolarić Kravar, Verica Kralj, Ivan Pećin, Lea Pollak, Dunja Skoko-Poljak, Danijela Stražanac, Ana Stupin, Vanja Vasiljev, Valentina Vidranski, Željko Reiner

**Affiliations:** 1 Department for Nephrology, Hypertension, Dialysis and Transplantation, University Hospital Center Zagreb, 10000 Zagreb, Croatia; ana.jelakovic9@gmail.com; 2 Croatian Hypertension League, 10000 Zagreb, Croatia; 3 School of Medicine, University of Zagreb, 10000 Zagreb, Croatia; ivanpecin@yahoo.com; 4 Faculty of Medicine, University of Rijeka, 51000 Rijeka, Croatia; lobilajac@gmail.com (L.B.); vanjav@uniri.hr (V.V.); 5 Ministry of Agriculture, 10000 Zagreb, Croatia; marija.b-sermek@mps.hr (M.B.S.); sanja.k-kolaric@mps.hr (S.K.K.); 6 Faculty of Health Studies, University of Rijeka, 51000 Rijeka, Croatia; 7 Teaching Institute of Public Health, 51000 Rijeka, Croatia; 8 Croatian Institute of Public Health, 10000 Zagreb, Croatia; marija.bubas@miz.hr (M.B.); kcapak@hzjz.hr (K.C.); verica.kralj@hzjz.hr (V.K.); 9 Ministry of Health, 10000 Zagreb, Croatia; dunja.skoko-poljak@miz.hr; 10 Croatian Agency for Agriculture and Food, 31000 Osijek, Croatia; vlatka.buzjak.sluzek@hapih.hr (V.B.S.); andrea.gross-boskovic@hapih.hr (A.G.B.); danijela.strazanac@hapih.hr (D.S.); 11 Department of Physiology and Immunology, Faculty of Medicine Osijek, University of Osijek, 31000 Osijek, Croatia; idrenjancevic@mefos.hr (I.D.); anacavka@mefos.hr (A.S.); 12 Scientific Centre of Excellence for Personalized Health Care, University of Osijek, 31000 Osijek, Croatia; 13 Department of Oncology and Nuclear Medicine, University Hospital Center Sestre Milosrdnice, 10000 Zagreb, Croatia; tomislav.jukic@kbcsm.hr; 14 Department for Metabolic Diseases, University Hospital Center, 10000 Zagreb, Croatia; zreiner@kbc-zagreb.hr; 15 Department for Food Supplements, Biologically Active and Psychoactive Substances, Croatian Institute of Public Health, 10000 Zagreb, Croatia; lea.pollak@hzjz.hr; 16 Department of Clinical Chemistry, University Hospital Center Sestre Milosrdnice, 10000 Zagreb, Croatia; vvidranski@gmail.com; 17 Department of Cardiology and Congenital Diseases of Adults, Polish Mother’s Memorial Hospital Research Institute, 93-338 Lodz, Poland

**Keywords:** salt, sodium, potassium, iodine, epidemiology, public health, blood pressure, hypertension, cardiovascular mortality, non-communicable diseases

## Abstract

The World Health Organization recommends adjusting salt intake as a part of the nine global targets to reduce premature mortality from non-communicable chronic diseases as a priority and the most cost-effective intervention. In 2006, the main aim of the Croatian Action on Salt and Health was to decrease salt intake by 16% because of its critical intake and consequences on human health. We have organized educative activities to increase awareness on salt harmfulness, define food categories of prime interest, collaborate with industries and determine salt intake (24 h urine sodium excretion). It was determined that the proportion of salt in ready-to-eat baked bread should not exceed 1.4%. In the period 2014–2022, salt in semi-white bread was reduced by 14%, 22% in bakery and 25% in the largest meat industry. Awareness of the harmfulness of salt on health increased from 65.3% in 2008 to 96.9% in 2023 and salt intake was reduced by 15.9–1.8 g/day (22.8% men, 11.7% women). In the last 18 years, a significant decrease in salt intake was achieved in Croatia, awareness of its harmfulness increased, collaboration with the food industry was established and regulatory documents were launched. However, salt intake is still very high, underlying the need for continuation of efforts and even stronger activities.

## 1. Introduction

The average sodium intake worldwide is estimated at 4310 mg/day (10.78 g salt per day), which is significantly higher than physiological needs (500 mg/day) and more than double the amount the World Health Organization (WHO) recommends (2000 mg/day) [1]. Non-communicable diseases (NCDs) are the leading cause of death worldwide, responsible for 41.5 million deaths in 2016 (71% of the 57 million deaths), more than all the other causes combined [2]. Cardiovascular diseases (CVDs) account for most deaths and raised blood pressure (BP) is the leading risk factor for CVDs. The recent Global Burden of Disease Study (GBDS) showed that high BP accounted for 10.4 million deaths and 218 million disability-adjusted life years (DALYs) in 2017 [3]. According to the meta-analysis, the number of people with arterial hypertension (AH) doubled between 1990 and 2019 and currently is approximately 1.3 billion [4]. Even a small increase in BP is associated with an increase in CV risk, including stroke, myocardial infarction, coronary heart disease and heart failure [5]. The WHO 2023 Global report on AH stated that elevated BP is one of the most important risk factors for disability and death worldwide [6]. High salt intake, which is a well-established cause of high BP, was responsible for 3.2 million deaths and 70 million DALYs [1,7,8,9,10,11,12]. According to the GBDS from 2019, the number of deaths from CVDs associated with high salt intake is 41.08% higher than it was in 1990 [13]. A reduction of 100 mmol/day in 24 h urinary sodium excretion is associated with a systolic BP reduction of 5.56 mmHg [12]. While the primary health effect associated with a diet high in sodium is raised BP, there is a growing body of evidence documenting the impact of high sodium intake on various health outcomes, including gastric cancer [14,15], obesity [16,17,18,19,20] and osteoporosis [21]. Based on the analysis of the GBDS data, it was concluded that the total number of deaths from chronic kidney disease (CKD) associated with increased salt intake was 45,530, while the number of DALYs was 1.32 million [22]. In a prospective study, we found that in our population with low potassium intake, increased salt intake is a significant risk factor for new-onset CKD [23]. A 2021 Cochrane review concluded that reducing high salt intake in patients in the early stages of CKD and with albuminuria is associated with a reduction in BP, which may lead to a slowing of CKD progression and a reduction in CV risk [24]. Recently published research has confirmed that high salt intake increases the risk of type 2 diabetes [25], and a Cochrane review published in 2023 concluded that reducing salt intake in patients with diabetes can significantly lower BP, preventing the onset and slowing the progression of diabetic kidney disease and that salt intake in patients with diabetes, whether or not they have AH and whether or not they already have signs of CKD, must be less than 5 g per day (less than 2 g of sodium) [26]. In 2013, the WHO recommended that all Member States reduce population salt intake by 30%, as a part of the nine global targets to reduce premature mortality from NCDs by 25% by 2025 [27]. Reducing salt intake in populations is among the most cost-effective interventions to reduce the burden of NCDs and is therefore considered a priority action for all countries (an average cost-effectiveness ratio of ≤I$100/DALY averted in low- and lower middle-income countries) [28,29,30,31]. The Sodium Country Score Card was established as part of the WHO’s efforts primarily to reduce dietary sodium intake, but this system will continuously monitor countries’ performance and continue to be enhanced with additional features [32,33]. In 2016, the WHO published the SHAKE Technical Package for Salt Reduction, to further stimulate Member States in carrying out salt reduction strategies through five key action areas: surveillance, harnessing industry, adopting standards for labeling, knowledge, and environment [34]. In 2017, the WHO recommended several sodium-related best buys policies and other measures as practical actions that should be undertaken immediately, to prevent CVD [35]. In 2023, the WHO launched the first Global Report on Sodium Intake Reduction investigating the progress of countries which are implementing sodium reduction policies and their impact on population and CVD [36]. Finally, salt reduction recommendation is an essential part of all relevant guidelines [37,38,39,40,41].

Since it is obvious that the primary goal of decreasing salt consumption for 30% by 2025 will not be achieved, the WHO called for accelerated actions in scaling up efforts to reduce populations’ sodium intake.

## 2. Prevalence of Hypertension and Cardiovascular Mortality in Croatia

According to recent data, in 2022, CVDs were the leading cause of death in Croatia (39.1%), of which more women died from CVDs than men (43.8% vs. 39.1%) [42]. The age-standardized death rate in Croatia is significantly higher than the EU average (591/100,000 vs. 344/100,000). It is particularly worrying that the age-standardized death rate in people under 65 (57/100,000 vs. 41/100,000) is higher in Croatia compared to the EU average. Ischemic heart disease is the leading cause of death (12.2% or 6925 deaths), and hypertensive disease is in the second place (9.2% or 5231 deaths), which indicates that AH is the main cause of death in Croatia. Elevated BP ranks second after smoking as an important risk factor for DALYs in Croatia [43]. Today’s data are better than in 2009, the year when 49.2% of people died from CVDs, but since 2019, a decrease in the reduction in CVD mortality has been observed, which is mostly the consequence of the high prevalence of AH (50.9%). However, there are still many untreated patients with AH (23%), and control is achieved in only 50.1% of treated subjects with AH [44]. All these data undoubtedly confirm that AH is the main public health problem in Croatia. This is significantly contributed to by the large intake of salt in Croatia, which was recognized as early in 2006 [45].

## 3. Brief History of Eighteen Years Old Salt War in Croatia

### 3.1. First Period 2005–2014

**Figure 1:**
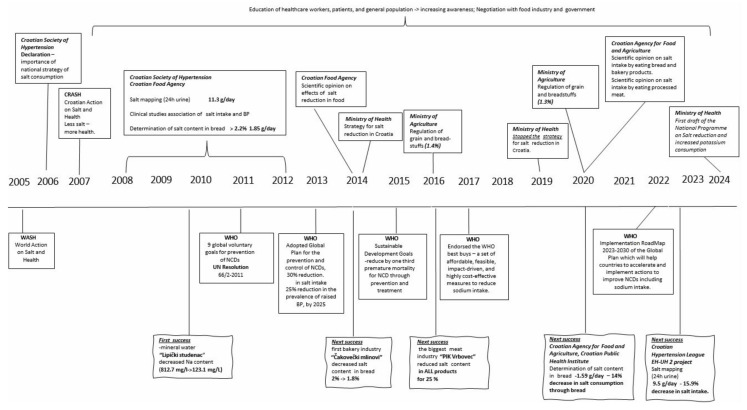
Chronology of the CRASH program in Croatia.

One year after the announcement of the World Action on Salt and Health (WASH) program, a Declaration on the importance of starting a national campaign to reduce salt intake in Croatia was accepted at the Congress of the Croatian Society for Hypertension in 2006, and in 2007 the Croatian initiative (Croatian Action on Salt and Health—CRASH) and the national program were presented at the Congress of the Croatian Atherosclerosis Society [45,46,47,48,49,50]. The motto of the CRASH program is “Less salt, more health”. The chronology of activities is shown in Figure 1.

In the initial period from 2005 to 2014, the following were carried out: (1) The average intake of salt in gram/day was determined by measuring the amount of sodium in a 24 h urine in (mmol/day), which is today the “gold standard” for salt intake estimation. The average intake of salt was 11.3 ± 4.41 g per day (men 13.37 g, women 10.37 g) (Table 1) [51]. (2) The relationship between the amount of consumed salt and BP values in the Croatian population was established which can be seen in the review by Jelaković, B. et al. [51,52,53]. (3) It was found that there was insufficient awareness of the general population about the harmfulness of excessive intake of salt (Table 2) [54]. (4) The proportion of salt in bakery products has been determined, ranging from 1.56% in some types of bread to an average of 2.0% (Table S1) [55,56,57]. (5) The proportion of daily salt intake from bread, bakery, meat and dairy products, and snacks was determined in a national, representative sample (Tables S2 and S3) [58]. The average total intake of salt from bread and bakery products was 2.46 g per day, which is half of the recommended value of the WHO. Moreover, only because consuming bread and bakery products, 7.8% of respondents consumed larger amounts of salt than the recommended daily intake [59,60]. (6) Education was organized by symposia for health workers, as well as public health actions and the preparation of popular educational brochures, leaflets and posters for patients and the general population. (7) Negotiations with the food industry, restaurants and catering facilities have begun. (8) A document—Scientific opinion on the harmfulness of excessive intake of salt—was prepared [45]. These first steps have already led to initial results. Public awareness of the harmful effects of excessive salt intake and the importance of reducing it increased, and parts of the industry have started to reduce voluntarily the NaCl content in their products (e.g., Lipički Studenac mineral water, Čakovečki Mlinovi bakery industry).

### 3.2. Action Plan for Salt Reduction in Croatia, Ministry of Health 2014–2019

**Figure 2:**
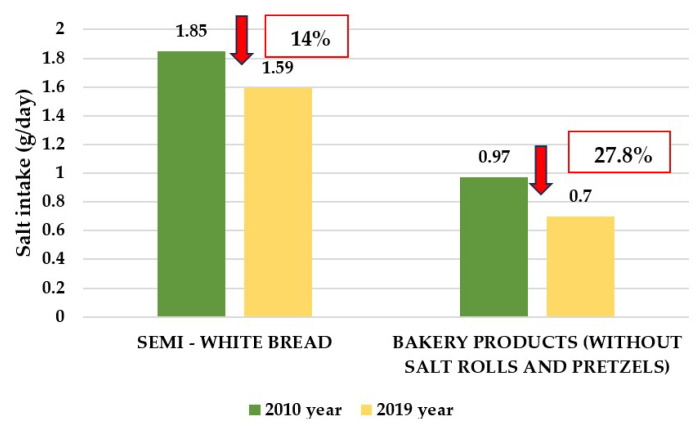
Decrease in daily intake of table salt through bread and bakery products at the beginning and the end of the third phase of the CRASH program.

These initial steps were the basis for the Action plan for salt reduction in Croatia 2014–2019, and it was prepared according to the WHO recommendations for reducing the intake of salt in the population: (1) To decrease salt intake by 16% over 4 years (4% per year in period of 2014–2019). (2) To increase awareness on salt harmfulness. (3) To define food categories of prime interest. (4) To determine salt intake by measuring 24 h urine sodium excretion. (5) To develop new recipes in collaboration with the food industry. (6) To monitor salt intake, collaboration with the food industry and analyze trends in awareness. In this period, the following were achieved: (1) In 2015, the Ministry of Agriculture adopted the Ordinance on Cereals and Cereal Products (OG 81/2016), which determined that the proportion of salt in ready-to-eat baked bread should not exceed 1.4% and in 2022, as second step, which determined that the salt content of ready-to-eat baked bread, as well as the baked pastry, may not exceed 1.3% [61]. With this Ordinance, Croatia joined a small number of European countries with such an advanced regulation (Table S4). (2) After years of negotiations, the largest meat industry in Croatia, PIK Vrbovec, made a reformulation of meat products and in 2016 reduced the proportion of salt in all its products by an average of 25%. We have continued with educative public health actions that increased awareness about salt (Table 2) [62]. (3) The control of the implementation of the project was performed by analyzing the share of salt in bakery products. In the analyzed bread samples, the amount of salt was on average 1.34% (0.9% to 1.8%) and in the bakery products 1.21% (0.7% to 1.7%). In semi-white bread, the amount of salt was reduced by approximately 14%, and by approximately 22% in bakery products (Figure 2) [59,63].

These results are consistent with another analysis, according to which the intake of salt by eating bread decreased by approximately 14% in the population, while this reduction in bakery products amounts to 28% [59]. According to the Croatian Institute of Public Heath (CIPH) study during 2019, the majority of the bakery industry was found to be compliant with the regulation (72% of breads and 66% of bakery products had a salt content <1.4%), which is consistent with data from the State Inspectorate and the Ministry of Agriculture, which found that the salt content in 89% of analyzed bread samples was compliant [59]. By contrast, study of the Faculty of Food Technology has shown that a significant number of small bakeries still do not comply with the regulations. Bread from large shopping centers, which is usually imported as a frozen form from other countries, which is not subject to the regulation, also contained a high content of salt [64]. (4) The success of the project implementation was analyzed by determining the urine salt excretion in 2008 and 2015. Unfortunately, the data obtained showed that the intake of salt had not decreased [65]. It should be noted that the study was conducted in a part of the rural population and that salt intake was estimated using spot morning urine samples, which is not the gold standard. Nevertheless, using the same method in the same population sample, we could conclude that, at least in this part of the population, there was no decrease in salt intake during this phase of the CRASH program, suggesting the need for a more intensive continuation of the program [50].

### 3.3. Third Period 2020–2024

Although officially the Action Plan of the Ministry of Health has not been continued, the Croatian Society for Hypertension, the Croatian Hypertension League, the Croatian Institute of Public Health (CIPH), the Croatian Agency for Agriculture and Food (CAAF), together with the Ministry of Agriculture, have continued with all previously planned activities. (1). In 2020, the Ministry of Agriculture adopted the Ordinance on Cereals and Cereal Products (OG 101/2022), which stated that the quantity of the salt in baked bread and bakery products ready for consumption should not exceed 1.3%. (2) The CAAF published two scientific reports: Scientific report on the intake of salt by the consumption of bread and bakery products, and Scientific report on the intake of salt by the consumption of meat products [58,59]. (3) The CIPH analyzed school meals.

The results showed that elementary school students still consume, only by eating at school, an excessive amount of salt (range 5.2–10.2 g per day). (4) Croatian Hypertension League and CIPH continued with numerous activities that increased the awareness of the general population. In this period, the Croatian Hypertension League further expanded these actions by launching the digital educational platform Hunting for the Silent Killer, where citizens are provided with various useful, important and practical information, which resulted in a significant increase in health literacy for this important segment of human health. Awareness of the harmful effects of excessive salt intake on human health increased from 65.3% in 2008 to 96.9%, and the number of people who believe that they could reduce their intake of salt increased from 69.4% to 87.9% (Table 2). (5) The intake of salt in the general population was determined by the “gold standard” method as a part of the Croatian Science Foundation project (Epidemiology of arterial hypertension and salt intake in Croatia—EH-UH 2) and public health actions organized by the Croatian Hypertension League and CIPH. According to the preliminary results, the intake of salt in the general population was reduced by an average of 15.9% for the total population, 22.8% for men and 11.7% for women (Table 1) [44]. (6) The CIPH conducted a project to analyze iodine in household salt, according to which 10% of salt samples did not contain the appropriate, prescribed amount of iodine (18–33 mg/kg as KI), in accordance with regulations [66]. The biochemical marker for assessing iodine is urinary iodine concentration (µg/L). According to the preliminary results of the EH-UH 2 project, where iodine intake was assessed by determining it in 24 h urine, the largest number of the adult population (54.5%) had adequate iodine intake (100–200 µg/L), insufficient intake (<100 µg/L) was found in 26.6% of the population, and 17.3% had an intake above the recommended level (>200 µg/L), which reflects the data of the CIPH on insufficiently adequate iodization or storage of salt, and possibly it also reflects the use of non-iodized salt, which is still preferred by a part of population [44].

## 4. Future Plans and Strategies—Decrease Salt and Increase Potassium Intake

During 2024, the Ministry of Health, together with partners who have been active all along, prepared a proposal for a Croatian National Prevention Program for reduction of excessive intake of salt with adequate iodine intake, stressing the importance of increasing potassium intake (Tables S5 and S6). A meta-analysis by Ma et al. showed that an increase in potassium excretion in 24 h urine for every 1000 mg was associated with an 18% lower CV risk [67]. Based on this and numerous other results, the WHO recommends that the minimum intake of potassium estimated by 24 h excretion should not be less than 90 mmol per day, which is equivalent to an approximate intake of potassium of 3.50 g per day. The WHO recommends increasing dietary potassium intake to reduce the risk of AH, CVDs and stroke [68,69,70]. According to the preliminary results of the EH-UH 2 project, the average intake of potassium in the general adult Croatian population is 2.98 g per day (men 3.17 g per day; women 2.86 g per day), which is below the WHO recommendation. The recommended intake of potassium estimated by a 24 h urine potassium excretion (≥90 mmol per day) was observed in 8.9% of subjects (10.9% of men and 7.8% of women). The average Na-to-K ratio was 3.1 (men 3.4 and women 3.0), which best reflects an unfavorable dietary pattern regarding the intake of salt and potassium. The percentage of people with a Na-to-K ratio ≤1, which is the WHO recommendation, was determined in a very small proportion of general population 3.2% (3.3% in men and 3.1% in women) [44]. A study on the quality of the diet of pregnant women found that a Na-to-K ratio of 2.68 (1.11–5.24) does not meet nutrition quality due to high sodium and insufficient potassium intake [71]. In our prospective study conducted in the general rural population, we found that the intake of salt above 10 g per day was present in 56.5% of respondents, and an intake of potassium >3.5 g per day in 3.2% with an extremely poor Na-to-K ratio of average 4.3 [72]. During an average of 7.5 years of follow-up, we observed that the Na-to-K ratio is a more important risk factor for new-onset AH than the intake of salt [73]. Based on all the evidence, the Guidelines of the European Society for Hypertension recommend non-pharmacological treatment of subjects with AH: (1). In subjects with AH who consume a high-salt diet, it is recommended to use substitute salts in which NaCl is replaced by KCl (evidence level I A). Increased intake of potassium, primarily through dietary changes, is recommended for all adults with elevated BP, except for patients in advanced stages of CKD [74].

These were the reasons for the added importance of an increase in potassium intake in addition to reducing the excessive intake of salt in the Croatian National Program. The new Croatian National Program continues to follow the SHAKE recommendations of the WHO [34] with the basic tasks: (1) the reformulation of food products to contain less salt and the setting of target levels for the amount of salt in foods and meals; (2) the establishment of a supportive environment in public institutions (hospitals, schools, kindergartens, workplaces, nursing homes) to enable lower sodium options to be provided; (3) a behavior change communication and mass media campaign; (4) the implementation of front-of-pack labelling. The Croatian National Program will have two arms—one aimed at increasing health literacy, which will be organized using classic public health methods, but also using digital technology (educational web platforms, social networks), and the other arm, which will be aimed at the food industry, restaurants, and catering.

## 5. Conclusions

The Croatian salt reduction initiative and activities (CRASH) have been very successful. In the general adult population, salt consumption was substantially reduced, and awareness about the harmful effects of high salt consumption significantly increased. A very good collaboration with the food industry has been established. This success might be a good lesson to other countries showing that improvements can be made even without the official support, endorsement and help of politicians. However, undoubtedly, these results would be even better with long-term multisectoral collaboration, and it is our hope that this will be achieved in the near future.

## Figures and Tables

**Table 1:** Salt intake (NaCl g/day) before the start of the CRASH program and at the end of the follow-up.

	2008	2020	Difference in Salt Intake	% Change
Whole group	11.3 (4.41)	9.5 (4.1)	−1.8 (3.8)	15.9 (3.9)%
Men	13.37 (4.34)	10.3 (4.4)	−3.0 (4.4)	22.8 (4.4)%
Women	10.37 (4.04)	9.0 (3.3)	−1.2 (3.1)	11.7 (3.2)%

Values are expressed as the mean (±standard deviation).

**Table 2:** Awareness of the harmful effects of excessive salt intake.

		World Hypertension Day, 2008 (%)	World Hypertension Day, 2017 (%)	May Measurement Month, 2023 (%)
N		1076	2175	10,480
Questions	Do you know that high salt intake is harmful for your health? Yes.	65.3	95.8	96.9
	Did you get this information from physician? Yes.	48.9	89.1	33.8
	Are you eating to salty? Yes.	27	36.1	36.2
	If advised, would you be able to reduce salt intake? Yes.	69.4	77.8	87.9

## Supplementary Materials

The following supporting information can be downloaded at: https://www.mdpi.com/article/10.3390/nu16101518/s1, Table S1: Salt intake at the beginning of CRASH program estimated from 24-hour sodium excretion and with spot urine sodium calculated by different equations; Table S2: The proportion of table salt in bread, bakery products and snacks before the start of CRASH program implementation; Table S3: The average daily intake of salt due to the average daily consumption by the type of food at the start of the CRASH program; Table S4: Regulation of table salt content in bread in the EU; Table S5: Areas and objectives of the Croatian National Program to reduce excessive salt intake and increase potassium intake in the period 2024–2028; Table S6: Targets and success indicators of the Croatian National Program to reduce excess salt intake and increase potassium intake in the period 2024–2028.
