# Supplementary material for: Croatian Action on Salt and Health (CRASH): On the Road to Success—Less Salt, More Health
Source: Nutrients. 2024 May 17;16(10):1518. doi: 10.3390/nu16101518 (PMC11124119; doi:10.3390/nu16101518)
Supplement: Supplementary file 1 [file nutrients-16-01518-s001.zip › nutrients-3004807-supplementary.pdf]

Supplementary materials

Table S1. Salt intake at the beginning of CRASH program estimated from 24-hour sodium excretion and with spot urine sodium calculated by different equations

	Salt mapping		Rural study	
	**24-hour urine**	**Kawasaki**	**Intersalt**	**Tanaka**
**Whole group**				
Sodium in urine (mmol/l)	201 (75.5)	216.2 (77.9)	2017.4 (51.21)	177.79 (60.86)
NaCl intake (gram/day)	**11.3** (4.41)	**12.64** (4.55)	**12.12** (2.99)	**10.39** (3.56)
**Men**				
Sodium in urine (mmol/l)	228.6 (74.2)	224.87 (78.52)	190.41 (39.28)	182.5 (48.00)
NaCl intake (gram/day)	**13.37** (4.34)	**13.14** (4.59)	**11.13** (2.30)	**10.67** (2.81)
**Women**				
Sodium in urine (mmol/l)	177.3 (69.1)	210.68 (77.03)	218.18 (54.86)	174.71 (67.84)
NaCl intake (gram/day)	**10.37** (4.04)	**12.31** (4.5)	**12.75** (3.21)	**10.21** (3.96)

The values are shown as the mean (± standard deviation)

Table S2. The proportion of table salt in bread, bakery products and snacks before the start of CRASH program implementation

**Type of product**	**Salt concentration (%) (average and range)**
Bread (23 types of bread)	1.56 (0.96-2.05)
**Bakery products**	
Little salty rolls (prstići)	2.73 (2.17-4.76)
Pretzel	2.98 (2.14-4.57)
Crescent roll (kifla)	2.37 (2.04-2.71)
Salty stick (štapić)	3.7 (2.51-5.98)
Salty roll (slanac)	2.41 (2.28-2.53)
Unsalted crescent roll	2.00 (1.89-2.1)
**Snacks**	
Bruschette	2.89 (2.41-3.3)
Salty sticks	3.43 (2.92-4.41)
Filled sticks	2.15 (1.7-2.8)
Salty pretzels	3.73 (2.85-5.65)
Salty snack "Ribice"	2.64 (1.59-3.16)
Crackers	2.79 (1.96-3.6)

Table S3. The average daily intake of salt due to the average daily consumption by the type of food at the start of the CRASH program

Type of food	Daily salt intake (g/day)
Ham	2.88
Pancetta	2.85
“Kulen” – Permanent sausage	2.55
Bread and bakery	2.45
Bacon	2.11
“Špek”	1.91
Prosciutto	1.87
Homemade sausage	1.76
Popcorn	1.36
Salty sticks snack	0.96
Rack	0.76
Salty crackers	0.69
Hard cheese	0.58
Potato chips	0.57
Corn flips	0.56

Table S4. Regulation of table salt content in bread in the EU

State	Regulated salt content<br>(g NaCl/100 g)<br>final product*/dry matter content**/added salt***	The average content of table salt on the market<br>(g NaCl/100 g bread)
**There is a regulation on cereals and bakery products**		
Croatia	1.3*<br>(Bread and Pastries)	0.9 -1.8<br>(0.7-1.7 pastries)
Slovakia	1.8 (Bread and Pastries, ***<br>(3.0 salty pastries)	1.11
Netherlands	1.8 **	1.0
Spain	1.66 *	1.15-1.30
Portugal	1.4*<br>traditional products with protected designations are excluded from the legal restriction	
Hungary	1.3 **	
Greece	1.5***	1.09-1.37
**There is no regulation on cereals and bakery products**		
Bulgaria	1.2*<br>defined in the Approved Standard	
France	1.4 plain bread*<br>1.3 whole meal bread or cereals*<br>1.2 sandwich bread*<br>Collective agreement of professionals in the bakery sector for a reduction in the quantity of salt in bread	
Slovenia	Study was conducted, to determine the average salt content of bread within national research programme Nutrition and Public Health. Scientific paper is still in preparation.	1.2<br>Salt content in prepacked bread part of continuous Slovenian monitoring program CLAS since 2011, Best-Remap project, in July 2022
Latvia	Memorandum of Cooperation on improving the composition of food products	1.0
Romania	1.3*<br>Standard from 1986.	1-1.2
Austria	Recommended by WHO	
Poland	1.02*<br>According to Polish food composition tables from 2017.	

Table S5. Areas and objectives of the Croatian National Program to reduce excessive salt intake and increase potassium intake in the period 2024-2028

Thematic area	Objective	Description
1. Increased awareness (health literacy) of the harmful effects of excessive salt intake in the population and the necessity of increased potassium intake, primarily through increased consumption of vegetables and fruit and then through the use of substitute salts with a higher content of potassium chloride.	1. To reduce the exposure of the population to the harmful effects of excessive intake of table salt and insufficient intake of potassium.	Raise awareness among professionals and the public about the dangers of excessive salt intake and inadequate potassium intake, the possibilities of prevention and the importance of its use as a treatment for people with NCD.
2. Increased partnership with the food industry and the hospitality sector.	1. Further reduction of the amount of table salt in bread and bakery products and start implementing measures to reduce table salt in snacks, dairy products, food supplements, convenience, and processed foods.	Reduce the proportion of table salt in bakery products by 0.1% every two years until the target value of 1.1% is reached in 2027 with the obligation to use iodized salt.
	2. Reduce the proportion of table salt in meat products, replacing sodium chloride with potassium chloride where technically feasible and requiring the use of iodized salt.	Reducing the proportion of table salt in all meat products by at least 25%, by replacing sodium chloride with potassium chloride where technically feasible.
	3. Increase the consumption of substitute salts containing potassium chloride, with the obligation that they are iodized.	Contributing to a simultaneous reduction of table salt intake and an increase in potassium intake with an adequate iodine intake, by using potassium chloride instead of sodium chloride.
	4. Reduce the preparation of food with excessive table salt in private and public restaurants (e.g. school canteens, school and hospital kitchens), including fast food chains, with a commitment to use iodized salt.	Contributing to the overall reduction of excessive table salt intake and the acquisition of healthy lifestyle habits by a diet that contains a smaller amount of table salt.
3. Establish a system for optimal monitoring and control of the implementation of the National program.	1. Monitoring the effectiveness of the implementation of the National Program.	Determining the success of the National program by assessing whether the predicted reduction in table salt intake by 16% and increase in potassium intake by 10% with adequate iodine intake was achieved in four years.

Table S6. Targets and success indicators of the Croatian National Program to reduce excess salt intake and increase potassium intake in the period 2024-2028.

Performance indicators	Initial values in 2024	Final value in 2027
Reduction of excessive salt intake in the general population	10 g per day	8.4 g per day (decrease 4% per year)
Increase the proportion of people with achieved salt intake targets ≤ 5 g per day (WHO recommendation)	10.5%	20%
Increase potassium intake in the general population	2.9 g per day	3.3 g per day
Increase the proportion of people with achieved potassium intake targets of ≥ 3.5 g per day (WHO and EFSA recommendations)	19%	30%
Increase the proportion of people with achieved target values for the Na/K ratio in 24 hours ≤1 (WHO recommendation)	3.2 %	15%
Reduction in the crude prevalence of arterial hypertension	50 %	40%
Increase the number of patients with arterial hypertension receiving controlled treatment	50%	70%
